# Breaking the outer membrane barrier: structure, targets, and antimicrobial strategies for Gram-negative bacteria

**DOI:** 10.3389/fmicb.2026.1734749

**Published:** 2026-02-24

**Authors:** Fanzhuo Xu, Yu Xie, Weiwei Yu, Zilin Wang

**Affiliations:** School and Hospital of Stomatology, Jilin University, Changchun, Jilin, China

**Keywords:** antimicrobial, bacteria, beta-barrel assembly machinery, efflux pumps, Gram-negative, lipopolysaccharide, membrane, nanocarriers

## Abstract

Multidrug resistance in Gram-negative bacteria has become a significant global public health challenge, threatening human health and clinical treatment outcomes. The unique outer membrane structure of these pathogens greatly limits antibiotic penetration, serving as the core mechanism of resistance. This paper systematically analyses antimicrobial strategies targeting the outer membrane of Gram-negative bacteria, mainly including: (1) directly disrupting the outer membrane structure and enhancing drug permeability; (2) inhibiting the biosynthesis or transport pathways of key outer membrane components; (3) using natural pathways to facilitate drug entry into the cell; (4) inhibiting efflux pumps to block efflux functions; (5) optimizing the physicochemical properties of drugs to enhance outer membrane permeability and using nanotechnology to develop new drug delivery systems. In recent years, BAM complex inhibitors like darobactin and xenorceptides have efficiently blocked the assembly of outer membrane proteins through a novel mechanism and exhibited excellent broad-spectrum antibacterial activity. Iron carrier-conjugated drugs like cefiderocol have also successfully transitioned to clinical use, showing significant efficacy in treating infections caused by various multidrug-resistant bacteria. Despite promising strategies targeting the outer membrane, drug development faces challenges, such as poor selectivity, potential toxicity, and evolving resistance mechanisms. Future research must delve deeper into the biosynthesis and regulatory mechanisms of the outer membrane, aiming to develop more selective and safer innovative antimicrobial drugs and delivery systems to effectively combat the growing threat of multidrug-resistant Gram-negative bacterial infections.

## 1 Introduction

Ranked as critically important pathogens by the World Health Organization (WHO), multidrug-resistant Gram-negative bacteria, specifically carbapenem-resistant *Acinetobacter baumannii* (*A. baumannii*), *Pseudomonas aeruginosa* (*P. aeruginosa*), and Enterobacteriaceae, represent a formidable barrier to modern medicine. These pathogens are capable of causing life-threatening infections, including hospital-acquired pneumonia, sepsis, and complicated urinary tract infections. Their ability to persist on medical devices and colonize environmental reservoirs makes them potent drivers of resistance dissemination in both clinical and community settings. The human cost of this resistance is staggering; according to recent estimates, approximately 4.71 million deaths worldwide in 2021 were associated with bacterial antimicrobial resistance. Notably, 1.14 million of these deaths were directly attributable to bacterial resistance, with *Escherichia coli* (*E. coli*) and *Klebsiella pneumoniae* (*K. pneumoniae*) among the leading pathogens contributing to these fatalities (GBD 2021 Antimicrobial Resistance Collaborators., 2022). The WHO’s updated 2024 bacterial priority pathogens list continues to emphasize the urgent threat posed by carbapenem-resistant Gram-negative bacteria and their central role in the global dissemination of antibiotic resistance (Sati et al., 2025).

The fundamental reason for the treatment challenges posed by Gram-negative bacteria lies in their unique cell envelope structure and multi-layered resistance mechanisms. Their cell envelope consists of an inner membrane, a periplasmic space containing a thin layer of peptidoglycan, and a highly asymmetric outer membrane, with its outer leaflet primarily composed of lipopolysaccharide (LPS). The outer membrane provides the cell with a strong barrier function, effectively blocking most antibiotics from entering the cell. This structural characteristic significantly enhances the intrinsic resistance of Gram-negative bacteria. Even if antibiotics successfully penetrate the outer membrane, Gram-negative bacteria can still rapidly expel them from the cell via active efflux pump systems (Gaurav et al., 2023). Additionally, bacteria often express various antibiotic-inactivating enzymes, such as β-lactamases and aminoglycoside-modifying enzymes, further weakening the efficacy of antimicrobial drugs (Reygaert, 2018).

Currently, treatment options for carbapenem-resistant Gram-negative bacterial infections are extremely limited (Cerundolo et al., 2025). Clinically, highly toxic drugs, such as polymyxins, or combination therapies whose efficacy has not been widely validated, are often required. The failure rate of treating resistant Gram-negative bacterial infections is high, and patient tolerance is poor, creating an urgent need for the development of new antimicrobial strategies and drugs.

The outer membrane not only constitutes an important barrier to drug penetration but also plays a crucial role in maintaining bacterial survival and environmental adaptability through its structure, composition, and biosynthesis. LPS and outer membrane proteins (OMPs) in the outer membrane are involved in nutrient uptake, signal transduction, and the regulation of pathogenicity, and are highly conserved across different bacterial species, making them important potential targets for the development of broad-spectrum antibiotics (Reygaert, 2018). Moreover, as these outer membrane components are absent in eukaryotes, targeting the outer membrane offers the potential for high selectivity and reduced toxicity risks. Strategies targeting the outer membrane not only aim to eliminate the pathogen itself but also have the potential to weaken its virulence.

Given the critical role of the outer membrane in resistance mechanisms and infection, this review will explore the intricate structure, molecular composition, and functions of the outer membrane in detail, analyze key molecular targets in its biosynthesis pathway, and discuss related targeted therapeutic strategies. We will also look ahead to emerging research directions, with the goal of providing scientific insight and guidance for addressing the growing threat of multidrug-resistant Gram-negative bacterial infections.

## 2 Role of the outer membrane in selective permeability and antibiotic resistance

The outer membrane of Gram-negative bacteria is a unique structure that forms the outermost layer of their cell envelope, distinctively different from that of Gram-positive bacteria. In the bilayer membrane structure, the outer membrane is located outside the thin peptidoglycan layer, forming an asymmetric lipid bilayer that provides these microorganisms with the crucial ability to survive and adapt in dynamic and even harsh environments. It not only acts as a physical barrier between the cell and the external environment, precisely regulating the entry and exit of substances, but also plays a central role in various life processes, including bacterial perception of environmental signals, nutrient acquisition, resistance to harmful substances including antibiotics and host defense molecules, and interactions with host cells.

### 2.1 Selective permeability barrier function of the outer membrane

The core physiological function of the Gram-negative bacterial outer membrane is to act as a selective permeability barrier, preventing the entry of harmful molecules such as antibiotics, detergents, bile salts, and host defense peptides while permitting the absorption of necessary nutrients. The asymmetric structure of the outer membrane lipids is key to this barrier function. This feature not only enables bacteria to survive and colonize successfully in complex host environments but also makes it difficult for larger antibiotics such as vancomycin and daptomycin to penetrate, thereby exhibiting intrinsic resistance (Gray and Wenzel, 2020; Maher and Hassan, 2023).

The outer membrane’s barrier function is dynamically regulated and can adapt to environmental stress, such as pH fluctuations, osmotic pressure changes, and antimicrobial peptide attacks. For example, bacteria can modify the surface charge of the outer membrane by partially adding phosphoethanolamine or Ara4N groups to the lipid A or core oligosaccharides of LPS via two-component regulatory systems like PmrA/PmrB (Maria-Neto et al., 2015). This alteration reduces the affinity for cationic antimicrobial peptides (CAMPs). Moreover, environmental factors such as osmotic pressure and nutrient concentrations can regulate porin expression. For instance, high osmotic pressure can promote the expression of OmpC while inhibiting OmpF expression (Maher and Hassan, 2023). This adaptive remodeling of the outer membrane, while beneficial for bacterial survival, may also influence antibiotic permeability and alter antibiotic sensitivity. Studying the dynamic regulation mechanisms of the outer membrane helps to uncover new resistance mechanisms and may provide insights for developing therapeutic targets that interfere with bacterial adaptability or restore drug sensitivity.

Although the LPS layer in the outer membrane forms a robust barrier, bacteria still need to acquire essential small molecules through porins in the outer membrane. Nonspecific porins allow hydrophilic small molecules such as monosaccharides, amino acids, peptides, nucleosides, and inorganic ions to passively diffuse into the periplasmic space. These channels are also the primary routes for many clinically important hydrophilic antibiotics, such as β-lactams, tetracyclines, chloramphenicol, and some fluoroquinolones, to enter bacterial cells (Overly Cottom et al., 2025). Therefore, the type, expression level, pore size, and charge selectivity of porins directly determine the rate at which antibiotics can enter bacteria. Reduced expression or structural mutations of porins can restrict antibiotic entry and significantly increase bacterial resistance.

### 2.2 Relationship between the outer membrane and antibiotic resistance

In the antibiotic resistance mechanisms of Gram-negative bacteria, the outer membrane barrier plays a fundamental and critical role. The barrier function of the outer membrane itself limits the entry of many antibiotics into the cell. The antibiotic resistance mediated by the outer membrane is primarily achieved through changes or loss of porins, overexpression or functional enhancement of efflux pump systems, and structural modifications of LPS (Kumari and Saraogi, 2025). For example, large molecules like vancomycin cannot penetrate the outer membrane, and therefore, Gram-negative bacteria have intrinsic resistance to them (Maher and Hassan, 2023). Similarly, hydrophilic β-lactam antibiotics must pass through outer membrane porins to enter the periplasmic space (Masi et al., 2022). If bacteria reduce porin expression or narrow the pore size through mutation, antibiotic uptake is significantly reduced. This entry barrier makes Gram-negative bacteria much less sensitive to large molecules and many hydrophilic antibiotics such as vancomycin and β-lactams, making them more difficult to affect by these antibiotics compared to Gram-positive bacteria (Maher and Hassan, 2023). The modification of LPS mainly impacts resistance to polymyxin antibiotics.

When this natural barrier is combined with mutations or regulatory changes in specific resistance genes, the level of bacterial resistance can increase significantly. For instance, in Enterobacteriaceae, a decrease in the expression of OmpC and OmpF is closely associated with resistance to β-lactam antibiotics, particularly when coexisting with β-lactamases (Davin-Regli et al., 2024). Furthermore, the loss of the CarO porin in *A. baumannii* often leads to carbapenem resistance (Siroy et al., 2005).

Efflux pumps like AcrAB-TolC and MexAB-OprM are key contributors to multidrug resistance in Gram-negative bacteria. Efflux pumps recognize and expel a wide range of antibiotics, including β-lactams, fluoroquinolones, and tetracyclines (Lorusso et al., 2022). This reduces the intracellular drug concentration, preventing effective antibacterial or bactericidal activity. The upregulation or functional enhancement of efflux pumps can significantly lower intracellular antibiotic levels, often leading to clinical multidrug resistance.

These resistance mechanisms do not exist in isolation but rather act synergistically to build a robust resistance barrier. In clinically isolated resistant strains, multiple mechanisms are typically detected simultaneously, such as overexpression of efflux pumps, decreased outer membrane permeability, increased production of antibiotic-inactivating enzymes, and mutations in antibiotic target sites. These mechanisms work together to enhance the bacteria’s resistance to multiple antibiotics. This multifaceted defense strategy can be conceptualized as a series of sequential barriers that an antibiotic must overcome to reach its target.

## 3 Disrupting outer membrane integrity and enhancing drug permeability

Directly disrupting the integrity of the Gram-negative bacterial outer membrane is an important antimicrobial strategy. By weakening this critical permeability barrier, the permeability of other antibiotics can be enhanced, improving antimicrobial efficacy or directly leading to bacterial death. The unique asymmetric bilayer structure of the outer membrane, particularly the LPS abundant in its outer leaflet, is the primary target of these disrupting agents.

### 3.1 Chelating agents to remove divalent cations

The stability of the Gram-negative bacterial outer membrane largely depends on divalent cations (primarily Mg^2+^ and Ca^2+^), which bind electrostatically to the core region of the LPS molecules and the phosphate groups of lipid A, maintaining the structural integrity of the outer membrane. Chelating agents disrupt this stability by removing these critical divalent cations, thereby breaking the connections between LPS molecules and disrupting the outer membrane structure, increasing its permeability to hydrophobic substances and antibiotics (Stephan et al., 2023).

Ethylenediaminetetraacetic acid (EDTA) is a classic chelating agent whose mechanism of outer membrane disruption has been extensively confirmed. EDTA effectively chelates the divalent cations in the outer membrane, disrupting the electrostatic crosslinks within the LPS layer and leading to the partial dissociation of the LPS molecular structure. This action destabilizes the outer membrane structure, significantly increasing its permeability to various compounds, including antibiotics that typically struggle to penetrate Gram-negative bacterial cells. For instance, EDTA has been shown to increase the sensitivity of *Haemophilus influenzae* biofilms to ampicillin and ciprofloxacin (Pakkulnan et al., 2024). Additionally, EDTA disrupts the electrostatic interactions between these divalent cations and extracellular DNA (eDNA), destabilizing the eDNA matrix and reducing the mechanical strength of the biofilm matrix, thus effectively inhibiting biofilm formation (Raad et al., 2008; Wang D. et al., 2025).

Although EDTA demonstrates good outer membrane disruption and synergistic enhancement *in vitro*, its clinical application as a systemic therapeutic agent is significantly limited. This is primarily due to EDTA’s ability to chelate divalent cations essential for host cell functions, potentially causing host cell dysfunction (Vervaet et al., 2017). Its rapid clearance from the body and potential renal toxicity further restrict its clinical safety and efficacy (Griffin et al., 2019). In addition to EDTA, other chelating agents have also shown similar abilities to enhance outer membrane permeability and synergize antimicrobial activity. For example, sodium hexametaphosphate, a potent Ca^2+^ chelator, enhances the antimicrobial activity of hydrophobic antibiotics against Gram-negative bacteria and increases the uptake of N-phenyl-1-naphthylamine, a fluorescent probe used to measure outer membrane permeability, indicating its ability to enhance outer membrane permeability (Vaara and Jaakkola, 1989). Citric acid and 2,3-dimercaptosuccinic acid, which is used clinically to treat lead poisoning, have also been reported to synergize with various antibiotics, with the mechanism involving increased outer membrane permeability (Jacinto et al., 2025). These different types of chelating agents all disrupt the outer membrane through similar mechanisms, further highlighting the vulnerability of the Gram-negative bacterial outer membrane to its dependence on divalent cations. However, translating this effective *in vitro* mechanism into a safe clinical drug remains a significant challenge, and future research needs to focus on developing more targeted and less toxic antimicrobial strategies.

### 3.2 Polymyxins and their derivatives

Polymyxin class antibiotics, such as polymyxin B (PMB) and polymyxin E (colistin), are cyclic lipopeptides. These compounds are characterized by a decapeptide ring, composed of multiple positively charged L-α,γ-diaminobutyric acid (Dab) residues, along with a hydrophobic acyl chain, usually 6-methyl-octanoyl or octanoyl, attached to the peptide ring (Slingerland et al., 2022). The peptide ring structure of PMB and polymyxin E differs mainly in their acylation patterns, with PMB containing a D-Leu and polymyxin E containing a D-Phe at a key position, along with differences in their fatty acid side chains.

The main mechanism of action of polymyxins is their specific interaction with LPS on the outer membrane of Gram-negative bacteria. The positively charged Dab residues on the polymyxin molecule interact electrostatically and bind with high affinity to the negatively charged phosphate groups on the lipid A region of the LPS molecules (McInerney et al., 2016). This binding competitively displaces the divalent cations (Mg^2+^, Ca^2+^) that originally stabilize the LPS layer structure, leading to a disorganized arrangement of the LPS molecules and destabilization of the outer membrane. The disruption of the outer membrane increases its permeability, and the hydrophobic acyl chain of polymyxin can effectively insert into the damaged areas of the outer membrane, facilitating the entry of the molecule into the periplasmic space (Khadka et al., 2018). The polymyxin then interacts with the cytoplasmic membrane, causing damage to it and leading to leakage of cellular contents. Clinically, despite its significant nephrotoxicity and neurotoxicity, polymyxin is still used as a last-resort drug to treat multidrug-resistant Gram-negative bacterial infections, particularly carbapenem-resistant strains, when other antibiotics fail (Ballı et al., 2024).

**FIGURE 1:**
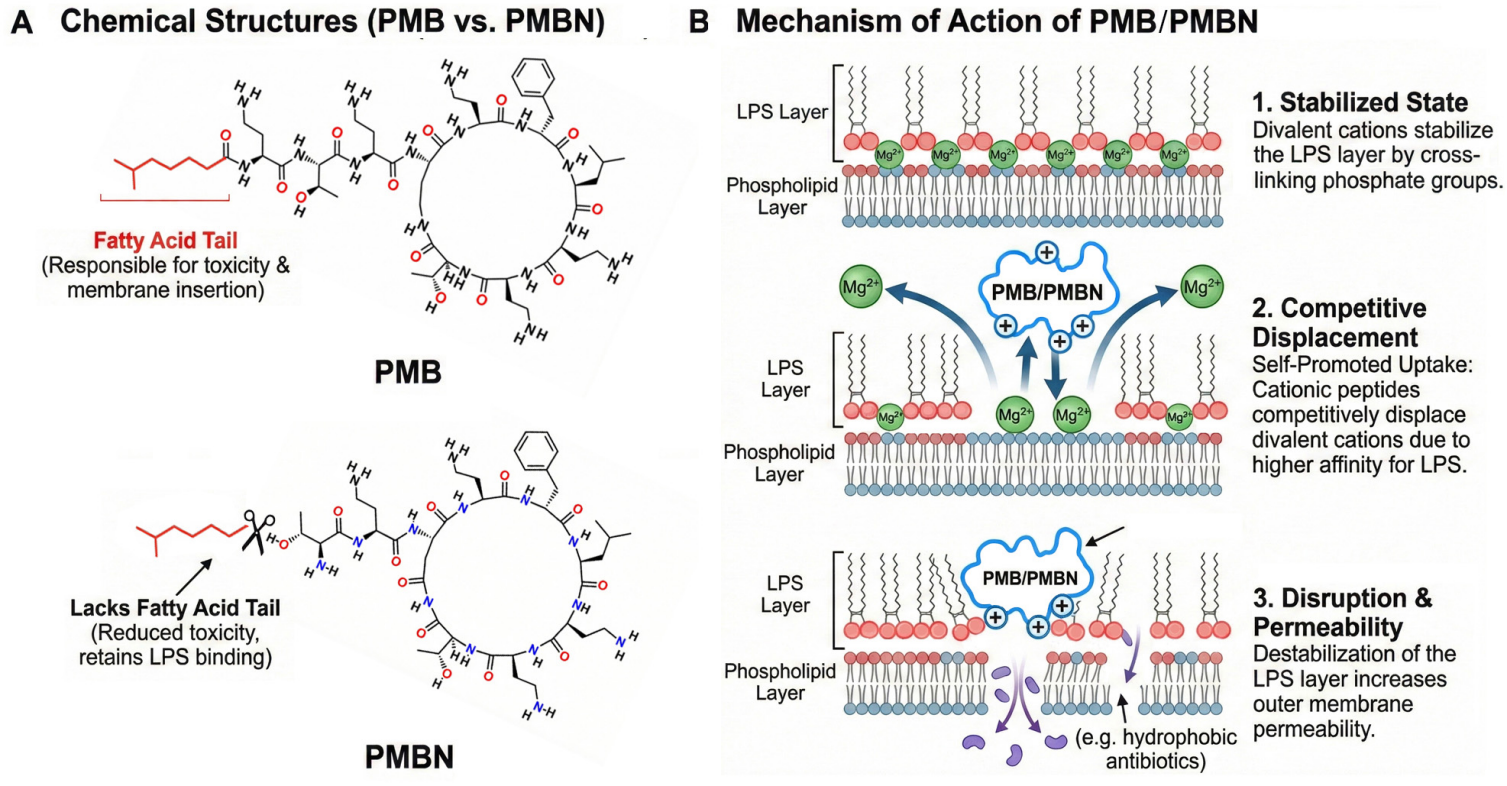
Comparison of chemical structures and membrane permeabilization mechanism of PMB and PMBN. **(A)** Chemical structures showing the presence of the toxicity-associated hydrophobic fatty acid tail in PMB and its absence in PMBN which retains the cyclic peptide ring required for LPS affinity. **(B)** Mechanism of outer membrane disruption. The electrostatic interaction of cationic PMB/PMBN with negatively charged phosphate groups on Lipid A competitively displaces the stabilizing divalent cations (Mg^2+^). This disrupts the integrity of the LPS layer, increasing outer membrane permeability and allowing the passage of hydrophobic antibiotics (indicated by purple arrows).

To overcome the toxicity issues of polymyxins while retaining their ability to disrupt the outer membrane, researchers have developed various derivatives. One important milestone is polymyxin B nonapeptide (PMBN). PMBN is a derivative of PMB obtained by the proteolytic cleavage of its N-terminal fatty acid-acylated tripeptide side chain. This structural modification removes the hydrophobic tail required for inner membrane insertion, thereby abrogating direct bactericidal activity and significantly reducing toxicity (Tsubery et al., 2001). However, PMBN retains the polycationic cyclic peptide structure, enabling it to bind avidly to anionic Lipid A. This interaction competitively displaces the divalent cations (Mg^2+^, Ca^2+^) essential for stabilizing the LPS leaflet, leading to outer membrane disorganization (Figure 1). Consequently, PMBN serves as a powerful outer membrane permeabilizer. By compromising membrane integrity, it acts as a potentiator for antibiotics generally ineffective against Gram-negative bacteria, such as hydrophobic rifampin, macrolides, and certain beta-lactams (Slingerland et al., 2022). Furthermore, studies indicate that PMBN not only lowers the minimum inhibitory concentration (MIC) of these agents synergistically but also improves efficacy against bacterial persister cells and reduces the frequency of resistant mutant strains (Wesseling and Martin, 2022).

### 3.3 CAMPs and their mimics

Antimicrobial peptides (AMPs), particularly CAMPs, represent a promising new generation of antibiotics. Naturally occurring CAMPs are a key component of the host defense system and possess broad-spectrum antimicrobial activity. They are typically cationic amphipathic molecules consisting of 12–40 amino acids, rich in cationic amino acids such as lysine and arginine, and hydrophobic amino acids. AMPs exert their antibacterial effect by interacting electrostatically and hydrophobically with bacterial membranes, especially the outer membrane of Gram-negative bacteria, which is rich in LPS, and the inner membrane, which is rich in phosphatidylglycerol or cardiolipin (Gagandeep et al., 2024). This interaction destabilizes the membrane structure, forms transmembrane pores, and dissipates the membrane potential, thereby increasing the membrane’s permeability to other molecules and ultimately leading to bactericidal activity.

Antimicrobial peptides typically act rapidly and are less likely to induce bacterial resistance. This is because they can nonspecifically disrupt bacterial membrane structures or target multiple sites simultaneously, making it difficult for bacteria to develop resistance through single mutations or simple metabolic changes (Yang et al., 2024). In addition to natural CAMPs, numerous synthetic peptide mimics with optimized characteristics, such as enhanced stability, higher antimicrobial activity, and lower host toxicity, are also being developed. These mimics aim to replicate the key physicochemical properties of natural CAMPs while overcoming certain drawbacks (Yadav and Chauhan, 2024).

A typical example of a natural AMP is LL-37, which is produced by the human body. LL-37 not only has direct antimicrobial and antibiofilm activity but also has immunomodulatory functions, modulating chemokine expression, bidirectionally regulating inflammatory responses depending on context, enhancing macrophage phagocytosis, and stimulating the release of inflammatory mediators (Pahar et al., 2020). Its antimicrobial mechanism primarily involves binding to bacterial membranes and perturbing their structural integrity, possibly leading to transient pore-like disruptions or membrane destabilization, as well as targeting multiple intracellular sites (Chen et al., 2023). In addition to membrane disruption, some AMPs can translocate into the cytoplasm and interfere with key intracellular processes, such as inhibiting DNA, RNA, or protein synthesis and enzymatic activity; although interference with protein folding has also been suggested, the underlying mechanisms remain unclear. For example, buforin II and indolicidin target nucleic acid or protein synthesis (Cardoso et al., 2019). Many AMPs possess multi-target characteristics, acting on both the cell membrane and intracellular targets. This multi-target feature substantially lowers the likelihood of rapid resistance development, although bacterial resistance to AMPs can still emerge under prolonged or sub-lethal exposure through mechanisms such as surface charge modification, efflux pumps, or proteolytic degradation.

Moreover, compared to traditional antibiotics that target a single molecular site, bacteria find it harder to evolve resistance against membrane physical disruption. AMPs offer unique advantages and great potential in treating resistant bacterial infections, as they are less likely to induce resistance (Zhu et al., 2025). However, their clinical translation faces numerous challenges, including insufficient understanding of their detailed mechanisms of action, lack of universal rational design principles, complex drug delivery routes, potential host cell toxicity, immunogenicity risks, poor *in vivo* stability, and high production costs (Cresti et al., 2024). Current research focuses on improving the stability, antibacterial activity and reducing the toxicity of AMPs through peptide engineering or synthetic mimics. Future development may focus more on developing peptide mimics that replicate AMP penetration mechanisms but offer higher *in vivo* stability, lower host toxicity, and greater economic feasibility, such as stabilized-modified peptides or novel non-peptide small molecules (Mwangi et al., 2023). For example, fusing bacteriolytic lysins that can degrade bacterial peptidoglycan with AMPs could significantly enhance their activity against Gram-negative bacteria (Abdelkader et al., 2022; Son et al., 2023). Some peptide-based permeabilizers, such as SPR741, a non-antibacterial derivative of PMB, are being developed as adjuvants to enhance outer membrane permeability and facilitate the efficacy of co-administered antibiotics. Several AMPs or their derivatives, including PL-5, DPK-060, brilacidin, and LTX-109, have already entered preclinical or various stages of clinical trials (Gan et al., 2021).

## 4 Inhibition of outer membrane biosynthesis

In addition to directly attacking the existing outer membrane structure, another promising strategy is to block the biosynthesis and transport processes of its key components from the source, thereby preventing the formation of a functional outer membrane barrier. Targeting the key biosynthetic pathways responsible for assembling outer membrane components offers a promising avenue for developing new antibiotics, as these pathways are typically unique to bacteria and are crucial for their survival.

### 4.1 Targeting LPS

As the major component of the outer leaflet of the outer membrane, LPS functions as a potent endotoxin, enabling bacteria to resist external stressors and contributing to immune system activation during infection. Therefore, inhibiting LPS biosynthesis or blocking its transport from the inner membrane to the outer membrane can effectively disrupt the structural integrity of the Gram-negative bacterial outer membrane, thereby reducing bacterial survival. This represents an effective approach for the development of new antimicrobial drugs.

#### 4.1.1 Targeting LPS biosynthesis

The minimal functional unit of LPS isKdo_2_-lipid A, which consists of lipid A and two 3-deoxy-D-manno-oct-2-ulosonic acid (Kdo) residues, is synthesized via the highly conserved Raetz pathway in Gram-negative bacteria (Wang et al., 2015). Lipid A serves as the hydrophobic anchor structure of LPS and is critical for the integrity of the outer membrane. The key enzyme in this pathway is LpxC, a zinc-dependent metalloenzyme that catalyzes the committed and rate-limiting deacetylation of UDP-3-O-acyl-N-acetylglucosamine during lipid A synthesis. Due to the absence of homologs in mammalian cells, LpxC has become a highly selective target for antimicrobial drug development (Ji et al., 2025).

Representative LpxC inhibitors include L-161240, discovered early by Merck, which has a MIC in the nanomolar range against *E. coli* and demonstrates significant antibacterial activity *in vitro* and in mouse infection models. Later-developed hydroxamic acid derivatives further broadened the antimicrobial spectrum (Krause et al., 2019). For example, BB-78485, a sulfonamide hydroxamic acid derivative developed by British Biotech Pharmaceuticals, exhibits broad-spectrum inhibition against Enterobacteriaceae, *Haemophilus influenzae*, *Moraxella catarrhalis*, and *Burkholderia cepacia* (Niu et al., 2023). CHIR-090, an aryl-substituted hydroxamic acid derivative discovered by Chiron Corporation, showed micromolar-level inhibitory activity against *P. aeruginosa* (Niu et al., 2023).

Although LpxC inhibitors have shown great antimicrobial potential, clinical development remains challenging. For example, ACHN-975, the first LpxC inhibitor to enter phase I clinical trials, was discontinued due to dose-dependent cardiovascular toxicity and severe injection site reactions. This toxicity may be related to the strong metal-chelating activity of the hydroxamic acid group in its structure, which may non-specifically chelate other intracellular zinc-dependent enzymes, such as histone deacetylases or matrix metalloproteinases, resulting in undesirable off-target effects and systemic toxicity (Cohen et al., 2019). Therefore, future LpxC inhibitors should avoid such groups and explore novel chemical backbones. Current research is primarily focused on structural optimization, structure-activity relationship studies, pharmacokinetics and toxicological evaluations, as well as the development of novel molecular backbones that avoid hydroxamic acid groups, such as thiol-, carboxylic acid-, or sulfonamide-based scaffolds, to reduce metal chelation-induced off-target effects.

#### 4.1.2 Targeting LPS transport

Once LPS is synthesized on the cytoplasmic side of the inner membrane, where lipid A and the core oligosaccharide are assembled, it must undergo transmembrane transport and insertion into the outer membrane’s outer leaflet to perform its function. This critical transport process is carried out by two essential molecular machine systems, namely the MsbA and Lpt systems (Sperandeo et al., 2017). Inhibiting these transport systems can effectively block the normal transmembrane transport of LPS and its assembly into the outer membrane, leading to impaired outer membrane barrier function and reduced bacterial survival (Romano and Hung, 2023). This presents a promising antimicrobial strategy.

MsbA is an ATP-binding cassette (ABC) transporter located on the inner membrane of bacteria, responsible for flipping newly synthesized lipid A-core oligosaccharide precursors from the inner leaflet to the outer leaflet of the inner membrane, representing the initial and essential step in LPS transport to the outer membrane (Bonifer and Glaubitz, 2021). Inhibiting MsbA activity results in the abnormal accumulation of LPS precursors on the inner membrane’s inner leaflet, disrupting normal outer membrane biosynthesis and ultimately leading to bacterial death. Reported MsbA inhibitors mainly include quinoline-based compounds such as G907, G592, G913 and G332, and tetrahydrobenzo[b]thiophene (TBT) compounds (Novischi et al., 2024). However, despite the effectiveness of quinoline-based inhibitors against multidrug-resistant strains, their clinical application is limited due to high serum protein binding, which not only significantly reduces the free drug concentration but also impairs tissue distribution and may alter drug efficacy or toxicity profiles. Therefore, future research should focus on developing new MsbA inhibitors with better pharmacokinetic properties or different binding sites (Romano and Hung, 2023).

The process by which LPS traverses the periplasmic space and is localized to the outer membrane is mediated by the Lpt system, a complex of multiple proteins that forms a transmembrane transport channel (Dajka et al., 2024). Inhibiting any key component of the Lpt system will obstruct LPS assembly and insertion, disrupting the integrity and function of the bacterial outer membrane. Murepavadin (POL7080) is a synthetic cyclic peptide antibiotic that specifically targets the LptD protein in the Lpt system of *P. aeruginosa*, demonstrating a narrow-spectrum activity. It blocks the insertion of LPS into the outer membrane’s outer leaflet, causing an accumulation of LPS precursors in the periplasmic space and leading to outer membrane damage (Moffatt et al., 2025). Murepavadin has shown excellent *in vitro* and *in vivo* antibacterial activity against *P. aeruginosa* and was once in phase III clinical trials (Díez-Aguilar et al., 2021; Moffatt et al., 2025). However, its systemic administration led to severe nephrotoxicity during the trial, causing the suspension of the phase III trial. The toxicity may be due to multiple factors, including the accumulation of the drug in renal tubular cells via renal excretion, potential off-target effects such as interference with mitochondrial SAM complex which is essential for the proper integration of β-barrel proteins into the outer mitochondrial membrane, and binding to the megalin receptor on renal tubular cells, leading to excessive endocytosis and exacerbated toxicity (Suzuki et al., 2013). Subsequent studies explored local administration via nasal or nebulized inhalation to treat *P. aeruginosa* infections in cystic fibrosis and non-cystic fibrosis bronchiectasis patients, reducing systemic exposure. Phase I clinical trials of the inhaled formulation showed favorable pulmonary pharmacokinetics and safety. Additionally, animal studies demonstrated that murepavadin increases bacterial cell membrane permeability and, when used in combination with aminoglycosides such as amikacin, exhibits synergistic bactericidal effects (Wei et al., 2024).

Another Lpt system inhibitor, zosurabalpin, is the first novel antibacterial drug targeting the inner membrane complex LptB2FGC. It shows significant antibacterial activity and efficacy in animal models against carbapenem-resistant *A. baumannii*, and both preclinical and phase I clinical studies have demonstrated its good safety profile (Zampaloni et al., 2024). Zosurabalpin has a novel mechanism of action and chemical backbone, and it is not affected by currently known resistance mechanisms, thereby offering a potential advantage over traditional antibiotics, meeting the WHO’s definition of an innovative antibiotic with significant clinical application potential. Although inhibitors targeting individual components of the Lpt system such as LptA or LptB subunits, have also been explored, they have failed to enter clinical stages due to toxicity or poor pharmacokinetic properties (Cardona et al., 2025). In contrast, zosurabalpin, which targets the LptB2FGC complex, shows better preclinical prospects, suggesting that future research on Lpt system inhibitors should focus more on using different chemical structures or targeting different subunits to avoid the toxicity and pharmacokinetic issues encountered in past development efforts, thereby further enhancing the clinical value of antimicrobial drugs (Zampaloni et al., 2024).

### 4.2 Targeting OMP assembly

#### 4.2.1 The BAM complex and its inhibitors

The β-barrel assembly machine (BAM) complex is a multi-protein complex located in the outer membrane of Gram-negative bacteria, responsible for correctly folding and inserting newly synthesized OMP polypeptides into the outer membrane. In *E. coli*, the BAM complex consists of five core components: BamA, BamB, BamC, BamD, and BamE, of which BamA and BamD are essential for bacterial survival (Fenn et al., 2024). BamA itself is an OMP with a transmembrane β-barrel structure consisting of 16 β-strands. The β1 and β16 strands form a lateral gate that can dynamically open and close, a structural change crucial for OMP insertion into the outer membrane (Tomasek and Kahne, 2021). BamD is a lipoprotein with multiple tetratricopeptide repeat domains, which directly interacts with the POTRA5 domain of BamA and may participate in recognizing unfolded OMP substrates (Fenn et al., 2024). Given BamA’s essential role, its high conservation, exposure on the cell surface, and its core function in OMP assembly, it has become a critical target for the development of new antibiotics.

In recent years, significant progress has been made in the development of inhibitors targeting the BAM complex, particularly the BamA subunit. Among these, darobactin and related bamabactin antibiotics have gained considerable attention. Darobactin, a natural product discovered from the bacterium *Photorhabdus khanii* of symbiotic nematodes, is a bicyclic peptide antibiotic (Imai et al., 2019). The representative compound darobactin A forms stable hydrogen-bond networks with the β1 strand of BamA, keeping the lateral gate closed and competitively inhibiting the OMP assembly process. Darobactins exhibit significant antimicrobial activity against various important Gram-negative pathogens, including *E. coli*, *K. pneumoniae*, *P. aeruginosa*, and *A. baumannii*, which are key ESKAPE pathogens, while showing almost no activity against Gram-positive bacteria (Böhringer et al., 2021).

On the basis of darobactin A, derivatives such as darobactin 22 have been developed, which bind more tightly to BamA and show better activity than the natural darobactin A. In mouse infection models including thigh infection, peritonitis/sepsis, and urinary tract infection models, D22 demonstrated superior *in vivo* efficacy and good safety. Additionally, its engineered derivative, darobactin D-sa, destroys the outer membrane integrity by inhibiting BamA function, leading to bacterial death. In a mouse lung infection model, darobactin D-sa significantly reduced the bacterial load of *P. aeruginosa* in lung tissues, indicating its potential clinical application in treating multidrug-resistant *P. aeruginosa* infections (Böhringer et al., 2021). Due to its novel targeting mechanism, darobactin antibiotics currently face no pre-existing resistance in clinical settings, highlighting their promising future as novel antimicrobial agents.

In addition to darobactins, other types of BamA inhibitors are also being explored. Recently, through genome mining and synthetic biology techniques, a new subclass of bamabactin, called xenorceptides, was identified, which induces complete remodeling of the transmembrane domain of BamA (Böhringer et al., 2021). Unlike darobactin, which stabilizes the closed state of the BamA lateral gate, xenorceptide A2 itself forms an additional β-strand that inserts between the β1 and β16 strands of BamA, expanding the original 16-strand β-barrel into a 17-strand hybrid β-barrel structure. This completely closes the lateral gate channel, preventing OMP substrates from entering the outer membrane, ultimately leading to bacterial death. Therefore, xenorceptides also exhibit potent bactericidal activity against Gram-negative bacteria.

In addition to bamabactin natural products, researchers are actively seeking BamA inhibitors through other approaches. For example, small molecule compounds such as MRL-494 and monoclonal antibody MAB1, which binds to the outer membrane loop 4 of BamA, also show specific antimicrobial effects, further validating the potential of BamA as a target (Walker et al., 2025). High-affinity cyclic peptides obtained via mRNA display technology have also been shown to inhibit OMP assembly and bacterial growth. The continued development of these different types of BamA inhibitors holds promise for overcoming current resistance challenges and providing new avenues and targets for the development of novel antimicrobial drugs.

#### 4.2.2 Targeting chaperones in OMP folding

The biosynthesis and proper folding of OMPs are critical for the function of the Gram-negative bacterial outer membrane, and periplasmic chaperone proteins play a key role in this process. Periplasmic molecular chaperones such as SurA and Skp function primarily as chaperones, whereas DegP exhibits both chaperone and protease activities. Together, they protect unfolded OMPs (uOMPs) after their release from the inner membrane Sec transport channel into the periplasmic space (Schiffrin et al., 2022). These chaperones prevent misfolding or aggregation of uOMPs and precisely deliver them to the outer membrane BAM complex. Among these periplasmic chaperones, SurA is considered the primary mediator of OMP transport, and interacts with the core subunit BamA of the BAM complex, possibly involving flexible structural regions and non-catalytic domains rather than solely relying on its PPIase domain, promoting the effective delivery and correct insertion of OMPs into the outer membrane (Fenn et al., 2024). Disruption or inhibition of SurA function significantly interferes with the folding and outer membrane integration of OMPs, causing uOMPs to be exposed in the periplasmic environment, increasing the risk of misfolding and aggregation, and ultimately leading to their recognition and degradation by the DegP protease. Such misfolding events reduce the effective insertion of OMPs into the outer membrane, compromising the integrity of the outer membrane, impairing nutrient uptake and signal transduction, and significantly increasing bacterial susceptibility to external stressors such as antibiotics and host immune defenses. Additionally, the deletion or inhibition of SurA has been shown to weaken bacterial virulence, as evidenced by defects in pilus assembly, diminished outer membrane barrier function, and reduced synthesis of iron carrier receptors (Soltes et al., 2016). Therefore, SurA has been identified as a potential target for anti-virulence strategies, as inhibiting OMP maturation and integration does not directly kill bacteria, but weakens their outer membrane barrier and virulence, thereby increasing susceptibility to host immune responses and co-administered antimicrobial agents.

Currently, researchers have identified several candidate small molecules with predicted binding affinity to SurA through virtual screening of the ZINC database, although most remain to be validated through biochemical or biophysical assays. Experimental studies have also found that Fmoc-β-(2-quinolinyl)-D-alanine and other Fmoc-modified amino acid derivatives exhibit some binding affinity for SurA, providing a preliminary structural framework for future optimization of SurA inhibitors (Bell et al., 2018). However, compared to the development of inhibitors directly targeting the BAM complex or LPS transport pathways, the development of inhibitors targeting SurA and other periplasmic chaperones is still in the early stages of exploration. This is primarily due to the complexity of SurA’s protein structure and the lack of a clearly defined, high-affinity ligand-binding pocket, which presents significant challenges for drug design. Nevertheless, a deeper understanding of the molecular mechanisms of SurA and other periplasmic chaperones will provide important theoretical support and practical guidance for the development of innovative antimicrobial strategies.

### 4.3 Targeting lipoprotein transport and maturation

Lipoproteins play a crucial role in maintaining the stability and functionality of the cell envelope, including processes such as nutrient uptake, signal transduction, outer membrane biosynthesis, and homeostasis. The biosynthesis, maturation, and localization of lipoproteins is a complex, multi-step process that is strictly regulated by a series of specific enzymes and transport systems. These pathways, characterized by highly conserved protein sequences and structures in bacteria, are essential for bacterial survival and lack homologous counterparts in eukaryotes. Thus, they have become important targets for the development of selective antibacterial drugs.

#### 4.3.1 Targeting lipoprotein transport

The Lol (lipoprotein outer membrane localization) system is responsible for the specific transport and localization of a subset of mature lipoproteins that are destined for the outer membrane, while others remain anchored in the inner membrane. This system consists of five core proteins: the ABC transporter LolCDE located in the inner membrane, the periplasmic chaperone protein LolA, and the receptor protein LolB located in the outer membrane. The transport process begins with the LolCDE complex in the inner membrane, which uses ATP hydrolysis to generate energy, promoting the detachment of the lipoprotein from the inner membrane and its binding to the soluble carrier protein LolA in the periplasm (Cole et al., 2021; Narita and Tokuda, 2010). The lipoprotein-bound LolA then crosses the periplasmic space and binds to LolB on the outer membrane, ultimately anchoring the lipoprotein to the inner leaflet of the outer membrane (Hayashi et al., 2014). Inhibition of any component in this system will prevent proper localization of lipoproteins to the outer membrane, leading to their abnormal accumulation in the inner membrane, disrupting outer membrane integrity, and ultimately causing bacterial death. Given the high conservation of the Lol system in pathogens and its indispensability for maintaining outer membrane structure, it has become a significant target in current antimicrobial drug development, with increasing focus on the development of specific inhibitors targeting key proteins in the Lol system.

Recent progress has been made in the development of inhibitors targeting the Lol system in Gram-negative bacteria. Early studies identified pyrimidinyl imidazole-based small molecules that interfere with the normal transport of the lipoprotein Lpp, marking the first reported inhibitors of LolCDE (Lehman et al., 2024). These compounds inhibit LolCDE functionality by binding to key sites on LolC or LolE, leading to lipoprotein transport defects, outer membrane structural abnormalities, and bacterial growth inhibition. However, these early compounds had poor membrane permeability, likely due to their high polarity or large molecular size, which hindered their ability to reach effective therapeutic concentrations within bacteria, thus limiting their efficacy in animal infection models and clinical potential. Recently, Muñoz et al. (2024) reported a novel macrolide antibiotic, lolamicin. Lolamicin competitively occupies the lipoprotein-binding site on the LolCDE complex, blocking normal lipoprotein transport. Experiments showed that lolamicin demonstrated significant selectivity and potent bactericidal activity against Gram-negative pathogens, with MIC ranges from 0.125 to 1 μg/mL against over 130 clinical multidrug-resistant strains (Muñoz et al., 2024). It also showed good therapeutic effects in mouse models of acute pneumonia and sepsis. Furthermore, lolamicin exhibited minimal activity against Gram-positive bacteria and intestinal commensals, and mouse model studies indicated that it did not significantly affect gut microbiota diversity and abundance, while effectively preventing secondary *Clostridium difficile* infections (de la Cuesta-Zuluaga et al., 2025; Zahra et al., 2025). This selective killing of pathogenic bacteria without affecting commensals is primarily attributed to structural differences in the LolCDE lipoprotein-binding domain and key amino acid variations between pathogenic and commensal bacteria, which determine lolamicin’s ability to selectively bind to pathogenic bacteria. Thus, lolamicin has become a representative compound for developing microbiome-friendly novel antibiotics targeting the Lol system.

Current research on Lol system inhibitors focuses on improving the membrane permeability and stability of existing compounds and developing novel inhibitors targeting co-factors of the Lol system, such as LolA or LolB, using small molecule screening, mRNA display technology, and monoclonal antibody approaches. Recent studies have identified pyridineimidazole compounds that inhibit the Lol system, specifically LolA-dependent release of lipoprotein Lpp, and are the first inhibitors of the LolCDE complex (McLeod et al., 2015). These studies offer new strategies for treating Gram-negative bacterial infections, but further evaluation is required to assess potential resistance mechanisms, such as the rate of target protein mutations and the possibility of functional replacement mechanisms. Additionally, the practical efficacy of these drugs should be comprehensively assessed under complex infection conditions, such as the presence of biofilms and host immune status, to determine their long-term clinical feasibility (Wang et al., 2023). Future research should also explore the synergistic mechanisms of Lol system inhibitors with other antimicrobial agents or host immune modulators to optimize combination therapies, aiming to enhance clinical efficacy against complex infections.

#### 4.3.2 Targeting lipoprotein processing enzymes

Before lipoproteins are transported to the outer membrane, they must undergo a critical maturation process on the inner membrane, which involves the coordinated catalysis of lipoprotein signal peptidase A (LspA) and lipoprotein N-acyltransferase (Lnt). LspA specifically recognizes and cleaves the signal peptide from pro-lipoproteins, thereby exposing a conserved cysteine residue. Following this, Lnt catalyzes the transfer of an acyl group derived from phosphatidylglycerol to the exposed cysteine residue at the N-terminal amino group, thereby completing the maturation process of lipoproteins (El Rayes et al., 2021). Both of these steps are crucial for the structural stability and function of lipoproteins. If either enzyme function is inhibited, immature lipoproteins accumulate abnormally in the inner membrane, causing cytotoxicity and ultimately leading to bacterial lysis (El Arnaout and Soulimane, 2019). Since LspA and Lnt are essential enzymes in Gram-negative bacteria, and their catalytic sites are located on the outer face of the inner membrane and lack homologs in human cells, they represent highly promising targets for antimicrobial drug development (El Arnaout and Soulimane, 2019). In recent years, with the rapid development of structural biology, the catalytic mechanisms of LspA and Lnt have been increasingly elucidated, providing an important theoretical foundation for structure-based rational drug design.

The natural product globomycin and its derivatives are currently widely used in the development of LspA inhibitors, and their mechanisms and pharmacological characteristics have been extensively studied. Globomycin binds non-covalently to the catalytic active site of LspA, effectively inhibiting its enzymatic activity and blocking lipoprotein maturation, exhibiting significant bactericidal activity in *E. coli* and other bacteria (Vogeley et al., 2016). However, globomycin has poor membrane permeability, making it difficult to effectively penetrate the outer membrane of Gram-negative bacteria and reach sufficient concentrations inside the cells for effective inhibition. Additionally, its potential non-specific toxicity to host cells further limits its clinical application potential. To overcome these challenges, researchers have modified globomycin’s structure and screened for new compounds to improve its outer membrane permeability and specific bactericidal effects. In one study, the derivative G0790 showed nanomolar-level inhibition of *E. coli* LspA and effectively inhibited bacterial growth (Pantua et al., 2020). Researchers have also attempted to develop structurally novel non-natural LspA inhibitors. Ayon (2023) used an *in vitro* high-throughput screening platform to identify a series of small molecule inhibitors and optimized their pharmacodynamics, resulting in a benzamide compound (Inhibitor-99) with potent non-competitive inhibition activity. Although this compound had low membrane permeability on its own, its combination with peptide-based permeabilizers significantly improved the drug’s membrane penetration ability, achieving intracellular concentrations sufficient to inhibit bacterial growth. These advances indicate that, in addition to natural products, developing small molecule LspA inhibitors with entirely new scaffolds also holds significant application potential.

Lipoprotein N-acyltransferase is another highly promising target for antimicrobial drugs. When Lnt activity is inhibited, lipoproteins remain in their diacylated immature state, unable to be effectively inserted or stably anchored in the outer membrane phospholipid layer, leading to significant disruption of membrane stability and ultimately affecting bacterial survival (Wiseman and Högbom, 2020). Lnt is an essential enzyme in many Gram-negative bacteria, such as *E. coli*, and its deletion results in abnormal lipoprotein processing and accumulation in the inner membrane, causing bacterial death (Noland et al., 2017; Song et al., 2025). However, it is worth noting that not all pathogens are absolutely dependent on Lnt. For example, *Francisella* spp., *Neisseria gonorrhoeae*, *Neisseria meningitidis*, *A. baumannii*, and *Helicobacter pylori* can maintain normal growth under laboratory conditions even in the absence of Lnt (Jung et al., 2023). This phenomenon suggests that drug development targeting Lnt should be tailored for specific pathogens to avoid insufficient or ineffective broad-spectrum strategies. These bacteria may compensate for the lack of Lnt by using alternative lipoprotein transport pathways, such as the LolF/LolD complex replacing the LolC/LolE complex to localize diacylated lipoproteins to the outer membrane. However, for major pathogens such as *E. coli* and *K. pneumoniae*, which are strictly dependent on Lnt, Lnt inhibitors still hold significant therapeutic potential (Wiseman and Högbom, 2020).

The natural product antibiotic myxovirescin (also known as antibiotic A) has been shown to be an effective inhibitor of Lnt. It forms an irreversible covalent complex with the Lnt substrate-binding channel, significantly inhibiting its acyltransferase activity. Studies have shown that myxovirescin and its semisynthetic derivatives exhibit potent bactericidal activity against several pathogens, including *E. coli* and *K. pneumoniae*, and they are less likely to induce resistance (Xiao et al., 2012). Recently, cryo-electron microscopy has further elucidated the structure of the Lnt-myxovirescin complex, providing important structural biological insights for structure-guided drug design and the development of new broad-spectrum antibiotics (Olatunji et al., 2020). These research findings highlight the targeting of lipoprotein maturation pathways as a promising strategy to combat antibiotic resistance. Future studies should further explore the synergistic effects of LspA and Lnt inhibitors with existing antibiotics to improve clinical outcomes against complex and resistant infections. Additionally, in-depth research into resistance mechanisms, such as mutations in target proteins and activation of alternative pathways, is crucial for the successful development of these inhibitors (Olatunji et al., 2020).

#### 4.3.3 Maintenance of lipid asymmetry (Mla) system

In addition to the biosynthesis of individual components, the overall homeostasis and asymmetric organization of the outer membrane is actively maintained by specialized systems, such as the maintenance of lipid asymmetry (Mla) system. The Mla system is a multi-component transport system widely present in Gram-negative bacteria, responsible for maintaining the compositional asymmetry of the outer and inner layers of the outer membrane. The Mla system consists of MlaA in the outer membrane which forms a complex with the porin OmpC/F, the lipid carrier protein MlaC in the periplasmic space, and the MlaFEDB ABC transport complex in the inner membrane (Coudray et al., 2020). Although early studies debated the direction of phospholipid transport mediated by Mla, recent biochemical findings have clearly indicated that the Mla system primarily functions to retrograde phospholipids, misplaced in the outer leaflet of the outer membrane, back to the inner membrane, thereby maintaining the structural integrity and functional stability of the outer membrane (Abellon-Ruiz, 2023).

Multiple studies have confirmed that defects in the Mla system significantly impair the barrier function of the bacterial outer membrane, increasing sensitivity to various antimicrobial agents, particularly large-molecule antibiotics and host defense components (Powers et al., 2020). For example, Mla-deficient strains of *Burkholderia cepacia* and *A. baumannii* show significantly increased sensitivity to macrolide antibiotics, rifamycins, and host immune factors such as complement in human serum (Powers et al., 2020). However, in laboratory strains of *E. coli* and *P. aeruginosa*, the impact of Mla system deletion on antimicrobial sensitivity is relatively weak, suggesting that the degree of dependency on the Mla system varies across different species (Ng and Moraes, 2023). Notably, in refractory opportunistic pathogens such as *A. baumannii* and *Moraxella catarrhalis*, the integrity of the Mla system is critical, and its functional defects severely disrupt the outer membrane barrier, significantly increasing bacterial sensitivity to antibiotics (Noel et al., 2024). Therefore, the Mla system has become a promising target for the development of novel antimicrobial drugs.

The novel cyclic lipopeptide antibiotic turnercyclamycin B (TCB) exhibits potent antimicrobial activity against a range of multidrug-resistant Gram-negative bacteria. Although Mla is not the direct target of TCB, its antimicrobial activity relies on a functional Mla system. Research has shown that bacteria achieve resistance to TCB through loss-of-function mutations in the mlaA gene or its regulatory regions, leading to the abnormal accumulation of outer membrane phospholipids in the outer leaflet, which in turn impedes TCB’s attack (Lim et al., 2023). This strategy of actively damaging the Mla system to resist drugs provides a novel perspective on antimicrobial resistance mechanisms. Moreover, a significant synthetic lethal interaction has been identified between the Mla system and the UppS enzyme in the undecaprenyl pyrophosphate biosynthesis pathway, providing a strong theoretical basis for combination therapy against multidrug-resistant bacteria such as *A. baumannii*. Studies have shown that a dual-hit caused by impaired uppS gene function and Mla system deficiency leads to catastrophic collapse of the cell envelope structure, severely weakening bacterial pathogenicity and survival (Noel et al., 2024). This synergistic effect has elevated the Mla system from a simple sensitization target to a potential combination therapy target, offering clear directions for the development of highly effective, low-resistance-risk combination treatment strategies.

With the high-resolution cryo-EM structure of the MlaFEDB complex and MlaC-MlaA/MlaD interactions, a new round of structure-based rational drug design is about to emerge. Future research should utilize high-throughput screening technologies such as CRISPRi and Tn-Seq to systematically explore other pathways and genes that exhibit synthetic lethal effects with the Mla system, further expanding combination treatment strategies against multidrug-resistant Gram-negative bacteria.

## 5 Promoting drug permeation across the outer membrane via natural entry pathways

The outer membrane of Gram-negative bacteria constitutes a natural barrier that greatly limits the efficiency with which antimicrobial agents penetrate the cell. To overcome this barrier, researchers have developed structural modification strategies for drugs, enabling antibiotics to utilize the bacteria’s inherent nutrient uptake channels or transport systems to penetrate the outer membrane. These strategies are primarily based on specific transport proteins and porin channels that bacteria have evolved to acquire essential growth nutrients such as iron carriers, carbohydrates and amino acids, as well as the promoting effects of electrostatic interactions and the outer membrane Donnan potential gradient on drug permeability. A deep understanding of and effective utilization of these natural molecular entry mechanisms are critical not only for enhancing the activity of existing antimicrobial drugs but also for providing important theoretical foundations and practical pathways for the design of highly effective, low-resistance new antibiotics.

### 5.1 Porin-mediated diffusion

Porins are the primary channels through which many small hydrophilic molecules, including antibiotics, enter the periplasmic space of Gram-negative bacteria. In general, porins allow small hydrophilic molecules with a molecular weight of approximately 600 Da or less to passively diffuse into the periplasm. This is the main route for the entry of β-lactam antibiotics, chloramphenicol, tetracyclines, and some fluoroquinolones across the outer membrane into the periplasmic space (Munita and Arias, 2016). When drug molecules exceed this size limit or exhibit strong hydrophobicity, their efficiency of permeation through porin channels is significantly reduced. Additionally, within the porin channel, there is a narrow region known as the “eyelet region,” which is composed of charged amino acid residues, creating a strong local electrostatic field that significantly affects the permeability of charged drugs. For example, the eyelet region of *E. coli* OmpF porin is rich in negatively charged amino acid residues, which generates a transverse electrostatic field that preferentially attracts and orients positively charged drug molecules, facilitating their translocation through the narrow channel (Maher and Hassan, 2023). However, when the charge of the drug molecule is repelled by the charge of the channel, its permeation efficiency is significantly reduced. Therefore, the structural size and electrochemical properties of porins jointly determine the effectiveness of drug passage through the outer membrane.

It is worth noting that the expression levels or structural mutations of porins are closely related to antibiotic resistance in Gram-negative bacteria. Under prolonged antibiotic selection pressure, pathogens often limit antibiotic entry by reducing porin expression or generating mutations in porin structure, thus significantly increasing resistance (Winterhalter, 2021). For example, downregulation or functional loss of OmpF or OmpC in *E. coli* can greatly reduce the ability of β-lactam antibiotics to enter the periplasmic space; *P. aeruginosa* decreases sensitivity to carbapenem antibiotics by downregulating OprD porin or undergoing structural mutations in the porin channel (Wang M. et al., 2025); similarly, impaired expression or function of the outer membrane porin CarO in *A. baumannii* shows a similar carbapenem resistance mechanism (Kyriakidis et al., 2021). These studies clearly show that the regulation and structural changes of porins are key mechanisms of antibiotic resistance in Gram-negative bacteria. Therefore, the development of antimicrobial drugs needs to fully consider the porin profile and expression patterns of the target bacterial species.

Given that porin-mediated diffusion is both an important pathway for antibiotic entry into Gram-negative bacteria and a key regulatory point for bacterial resistance, current research is increasingly focused on optimizing the size, charge distribution, and hydrophilicity of drugs to better match the porin channel structure and enhance permeability (Prajapati et al., 2021). Moreover, with the rapid development of computational simulation technologies, researchers widely use techniques such as molecular docking and molecular dynamics (MD) simulations to reveal, at the atomic level, the interaction mechanisms between antibiotics and porins. Ekaterina et al. combined *in vitro* single-molecule permeation experiments with *in silico* MD simulations to analyze the diffusion dynamics of cephalosporin antibiotics in *E. coli* OmpF and OmpC porins, elucidating the energetic barriers, key amino acid interactions, and conformational dynamics that govern whether a drug successfully translocates or becomes trapped within the eyelet region (Nestorovich et al., 2002). Additionally, the integration of virtual screening with machine learning is being used to build robust predictive models that can rapidly evaluate the porin permeability potential of novel drug candidates and uncover key structural features influencing permeability. These advanced methods provide in-depth mechanistic insights into porin-mediated drug diffusion, effectively guiding the rational design and structural optimization of novel antimicrobial drugs.

### 5.2 Specific transporter systems

#### 5.2.1 Siderophore receptors

Iron is an essential trace element for bacterial life activities, but in the host environment, iron is often bound to proteins such as transferrin and ferritin, presenting a state with low bioavailability. To effectively acquire environmental iron, most Gram-negative bacteria secrete small molecules called siderophores under iron-limiting conditions to chelate Fe^3+^, forming siderophore-iron complexes (Gräff and Barry, 2024). These complexes are actively taken up by specific TonB-dependent receptors located on the outer membrane. The receptor relies on the proton motive force provided by the TonB-ExbB-ExbD complex in the inner membrane to mediate the transmembrane transport of the iron complex (Ratliff et al., 2022). This highly specific and efficient nutrient uptake system provides a “Trojan horse”-like strategy for drug delivery in antimicrobial drug development.

One of the most successful clinical applications based on this strategy is cefiderocol. Cefiderocol is a siderophore-conjugated cephalosporin, which introduces catechol groups into the basic structure of cefepime/cefpirome to mimic bacterial natural siderophores (Bonomo, 2019). Through the catechol group, cefiderocol can bind with high affinity to the siderophore receptors of various Gram-negative pathogens such as *A. baumannii*, *P. aeruginosa*, and Enterobacteriaceae, and is actively transported across the outer membrane into the periplasmic space, overcoming the outer membrane barrier faced by conventional antibiotics (McCreary et al., 2021). Clinical studies have shown that cefiderocol exhibits potent antibacterial activity against drug-resistant strains, including those producing extended-spectrum β-lactamases, and was approved in Europe and the U.S. in 2019 for the treatment of serious infections such as complicated urinary tract infections and hospital-acquired pneumonia caused by *A. baumannii*, *P. aeruginosa*, and drug-resistant Enterobacteriaceae (Wang et al., 2024).

In recent years, other siderophore-conjugated antibiotics have also made significant progress. For example, the monobactam antibiotic BAL30072 structurally incorporates a hydroxypyridone group that mimics the natural siderophore function, thereby utilizing bacterial siderophore receptors to facilitate drug uptake (Page, 2019). BAL30072 has shown good antimicrobial activity *in vitro* against resistant strains such as *A. baumannii* and *P. aeruginosa*, and effectively reduced bacterial load in animal infection models. Additionally, similar strategies involving the modification of antibiotics with siderophore-inspired groups, such as enterobactin-conjugates of ciprofloxacin and fosfomycin, have been shown to enhance drug uptake in *Pseudomonas* via siderophore receptors, significantly improving antimicrobial efficacy (Khazaal et al., 2024).

Because the siderophore-mediated transport pathway is independent of porins and is generally not a substrate for many common efflux pumps, this drug delivery strategy can effectively bypass resistance mechanisms caused by reduced porin expression or overexpression of efflux pumps. It holds great potential for combating multidrug-resistant Gram-negative pathogens. This active transport mechanism provides an innovative pathway for the design of new antimicrobial drugs and indicates an important shift in the future direction of antibiotic development.

#### 5.2.2 Other nutrient transporter pathways

In addition to the siderophore receptor system, Gram-negative bacteria’s outer membrane also contains a wide array of specific transporters used for the uptake of nutrients such as carbohydrates, amino acids, and nucleosides. These transporters also have potential value as delivery vehicles for antimicrobial drugs. Among them, most transporters belong to the TonB-dependent transporter (TBDT) family, and their transmembrane transport process relies on the proton motive force provided by the TonB-ExbB-ExbD complex in the inner membrane (Celia et al., 2025). This characteristic theoretically makes TBDTs natural channels for antimicrobial drugs to enter bacterial cells.

Current research has explored chemically modifying antimicrobial drugs to resemble natural substrates, thereby utilizing these transport systems to actively enter cells. A successful example of this strategy is the natural compound Albomycin, whose structure is similar to the natural siderophore ferrichrome. Albomycin specifically binds to the ferrichrome receptor FhuA (formerly TonA) and is actively transported via the TonB system to enter the cell (Fujita et al., 2019). Additionally, some toxins produced by microorganisms, such as colicin and specific AMPs, can also be specifically recognized by bacterial outer membrane receptors through distinct energy-dependent pathways such as the Ton system (for siderophores) or the Tol system (for certain colicins), thereby entering the cell (Kim et al., 2014). This opens new perspectives and insights for antimicrobial drug development.

Although drug development based on non-siderophore nutrient transporter systems is still in the early stages, and no antibiotics utilizing these pathways have been clinically applied, preliminary studies have confirmed the potential of this strategy. For example, by covalently coupling antimicrobial molecules with maltose or nucleoside analogs, drugs can be specifically taken up by bacterial cells via the outer membrane maltose porin LamB or nucleoside transporter Tsx (Ropponen et al., 2021). However, compared to the siderophore system, these nutrient transporter systems are often more strictly and complexly regulated by metabolic mechanisms, and their transporters exhibit very high substrate specificity, which poses greater challenges for drug design (Zeden et al., 2021).

Future research needs to delve deeper into the structural recognition mechanisms between these TBDTs and their substrates. Using MD simulations and structural biology techniques, researchers should accurately predict the optimal binding sites between drugs and transporters, and critically evaluating the expression levels of these transporters under authentic infection conditions, to ensure that the designed ‘disguised’ antimicrobial drugs can find their entryways during clinical use. Furthermore, systematically assessing the activity expression patterns, substrate specificity, and related resistance mechanisms of different types of nutrient transporters will further promote the clinical translation and practical application of this innovative drug delivery strategy.

### 5.3 Electrostatic interactions and the Donnan potential

The permeability of the outer membrane of Gram-negative bacteria is influenced not only by the electrostatic gradient formed by ion distribution across the membrane but also by outer membrane proteins and lipid structure. The outer membrane surface, particularly the inner core of LPS and the lipid A region, is rich in phosphate groups, giving it an overall negative charge. Additionally, the O-antigen region of LPS also contributes to the charge distribution of the outer membrane. These negatively charged groups form electrostatic bridges with divalent cations on the membrane surface, stabilizing the structure of LPS molecules, which, in concert with the lipid bilayer and membrane proteins, helps maintain the integrity of the outer membrane (Clifton et al., 2016). Furthermore, this negatively charged environment creates a Donnan potential across the membrane, effectively attracting and concentrating positively charged cationic molecules, which increases their local concentration on the outer membrane surface, accelerating their passage through porin channels or direct binding with LPS (Alegun et al., 2021).

Polycationic antibiotics, such as aminoglycosides and polymyxins, exert their antimicrobial effects through electrostatic interactions with LPS, as well as other mechanisms such as binding to bacterial ribosomes. These antibiotics, due to their high cationic charge density and strong electrostatic affinity, can competitively displace the electrostatic bridges between LPS molecules and divalent cations, thereby destabilizing the outer membrane structure. This increases the outer membrane permeability, making it easier for antimicrobial molecules to enter the bacterial cell. A large body of research has shown that appropriately increasing the number of cationic groups in antibiotic molecules or optimizing the distribution of these charges can significantly enhance electrostatic interactions between the drug and the bacterial outer membrane, thus improving drug penetration efficiency. However, this strategy may carry risks of resistance development (Trimble et al., 2016).

However, it is important to note that the outer membrane permeability of a drug is not only determined by its charge properties but also limited by the molecule’s size and hydrophilicity. Since Gram-negative bacterial porin channels impose strict size limitations on molecules, merely increasing the positive charge of the drug molecule without addressing its size limitation may not achieve the desired cellular uptake (Kojima and Nikaido, 2013). Therefore, optimizing the combination of drug molecule charge distribution, size, and hydrophobicity is a key strategy to enhance outer membrane penetration efficiency.

Although improving drug penetration through electrostatic interactions offers significant advantages, excessively strong electrostatic interactions may disrupt the stability of the outer membrane lipid bilayer, damage membrane protein structures, and potentially trigger host inflammatory responses (Vejzovic et al., 2022). Therefore, in the development of novel antimicrobial agents, it is crucial to optimize the drug’s molecular structure to precisely balance its antimicrobial efficacy with potential toxicity risks, especially in the context of electrostatic interactions. Future research should further employ quantitative analysis and MD simulations to elucidate how drug charge distribution affects outer membrane permeability, and explore the optimal balance between improving antimicrobial efficacy and ensuring host safety in the process of drug structure selection and optimization, aiming to develop more efficient and safer new antimicrobial drugs.

## 6 Targeting efflux pumps

In addition to the formidable penetration barrier posed by the outer membrane, Gram-negative bacteria employ a second line of defense: powerful efflux pumps that actively expel antibiotics that have successfully traversed the outer membrane, further reducing intracellular drug accumulation. Efflux pumps play a key role in both intrinsic and acquired resistance by reducing intracellular drug concentrations, helping bacteria withstand antibiotic attacks and maintain their physiological balance. Therefore, inhibiting the activity of efflux pumps has become a potential strategy to enhance the effectiveness of antimicrobial therapy. This approach has been shown in several studies to be a promising adjunct therapy to restore the efficacy of existing antibiotics and slow the evolution of resistant strains.

### 6.1 Mechanisms and classes of efflux pumps

Gram-negative bacteria commonly possess a variety of multidrug efflux pumps composed of membrane transport proteins, which reduce intracellular drug concentrations and lead to resistance by actively expelling a wide range of harmful substances (Gaurav et al., 2023). Based on sequence homology, energy coupling mechanisms, and substrate specificity, bacterial efflux pumps are mainly classified into the ABC superfamily, major facilitator superfamily (MFS), multidrug and toxic compound extrusion (MATE) family, Resistance-Nodulation-Cell Division (RND) superfamily, small multidrug resistance (SMR) family, and proteobacterial antimicrobial compound efflux (PACE) family (Zack et al., 2024). Except for ABC superfamily pumps, which directly use ATP hydrolysis to drive efflux, all other types are secondary active transporters that rely on proton or sodium ion gradients for energy.

In Gram-negative bacteria, the RND-type efflux system is the most clinically important multidrug efflux pump, with typical representatives including *E. coli*’s AcrAB-TolC system and *P. aeruginosa*’s MexAB-OprM system (Blair and Piddock, 2009). These systems generally consist of three components: an RND transporter located in the inner membrane, a periplasmic membrane fusion protein such as AcrA or MexA, and an outer membrane channel protein such as TolC or OprM (Tikhonova et al., 2002). RND efflux pumps utilize proton motive force to induce conformational changes, working in coordination with membrane fusion proteins and outer membrane channel proteins to capture a wide range of substrates from the cell interior or periplasm and directly expel them through the outer membrane channel (Nikaido, 2011). Their substrate spectrum is extremely broad, efficiently expelling a variety of structurally diverse molecules, including tetracyclines, fluoroquinolones, chloramphenicol, macrolides, bile salts, and dyes. Due to the widespread presence and central role of RND efflux pumps in many important pathogens, they have become the primary targets for the development of efflux pump inhibitors (EPIs) (Duffey et al., 2024).

Apart from the RND family, Gram-negative bacteria also possess MFS and MATE-type efflux pumps. MFS-type efflux pumps typically exist as single-component, proton-dependent transporters and are relatively rare in Gram-negative bacteria, but, for example, the KpnGH pump in *K. pneumoniae* can effectively expel toxic substances such as dyes and disinfectants (Srinivasan et al., 2014). MATE pumps usually use Na^+^ or H^+^ gradients to drive transport; a representative member, PmpM in *P. aeruginosa*, can expel quaternary ammonium disinfectants, dyes, and some quinolone drugs (Zhang et al., 2023). The SMR family of efflux pumps has a compact and simple structure; for instance, EmrE in *E. coli* expels quaternary ammonium salts and dyes, among other hydrophobic cationic compounds (Bay and Turner, 2012). ABC superfamily efflux pumps use ATP hydrolysis as a direct driving force and mainly participate in the secretion of specific macromolecular toxins or certain antibiotics in Gram-negative bacteria, while broad-spectrum multidrug efflux is more commonly mediated by RND and MFS pumps (Zack et al., 2024). A typical ABC transporter is the *E. coli* MacAB-TolC system, which is involved in the export of macromolecules such as specific toxins and some macrolide antibiotics. The PACE family is a recently discovered group of efflux pumps, usually composed of a single protein, with a simpler structure than the complex multi-component RND efflux pumps. These pumps are widely found in *Acinetobacter* species and some Enterobacteriaceae, and can expel specific antimicrobial agents and disinfectants, such as chlorhexidine and PMB, thereby mediating low-level intrinsic resistance to these compounds (Chetri, 2023). Under environmental stress, the expression levels of PACE family members may increase and may synergize with other resistance mechanisms, such as RND pumps, to enhance overall resistance. Although no specific, highly efficient inhibitors of PACE family members have been reported, the mechanisms of action under certain conditions warrant further research, potentially providing a foundation for developing effective inhibitors.

### 6.2 Development of EPIs

The multidrug resistance of Gram-negative bacteria is largely attributed to their powerful efflux pump systems. Therefore, the development of EPIs to block efflux functions has become a potential adjunctive therapeutic strategy to restore the efficacy of existing antibiotics. EPIs increase the intracellular concentration of antimicrobial agents by inhibiting bacterial active efflux mechanisms, thereby enhancing the sensitivity of resistant strains to antibiotics (Pagès et al., 2005).

Although the importance of efflux pumps as antimicrobial targets is widely recognized, the path to translating effective EPIs into clinical applications is fraught with challenges, including issues such as pharmacokinetic properties, toxicity risks, and inconsistent efficacy *in vivo* and *in vitro*. Ideal EPIs should exhibit no independent antimicrobial activity, low toxicity, good outer membrane permeability, broad-spectrum inhibition of major efflux pump families such as RND, MFS, and MATE, and show significant synergy with various antibiotics (Rouvier et al., 2025). However, to date, no EPI has been approved for clinical use, primarily due to factors such as suboptimal pharmacokinetic properties of the compounds, high toxicity risks, and inconsistent efficacy *in vivo* and *in vitro*. Furthermore, prolonged use of EPIs could induce the upregulation of efflux pump expression or structural mutations, leading to the gradual development of resistance, further increasing the difficulty of development (Rouvier et al., 2025).

Classic small molecule EPIs are mostly synthesized or derived from existing drugs, typically working by competitively occupying the substrate-binding sites of efflux pumps or interfering with the energy supply required for pump function. An example is the dipeptide compound phenylalanine-arginine β-naphthylamide (PAβN), which effectively reduces the MIC of various antibiotics, including macrolides, fluoroquinolones, and chloramphenicol, by competitively binding to the substrate binding sites of RND efflux pumps (Sharma et al., 2019). However, the clinical translation of PAβN faces significant obstacles, including poor solubility, low stability, non-specific membrane permeability increase, potential toxicity, and poor pharmacokinetic properties. Therefore, current EPI development focuses more on optimizing molecular structure, improving drug selectivity, and enhancing pharmacokinetic properties to boost their clinical potential.

In recent years, research focus has gradually shifted toward the development of highly selective inhibitors targeting specific subcomponents of efflux pumps. For example, some novel inhibitors interfere with the functional rotation mechanism of the AcrB trimer or block the interaction between AcrA and AcrB/TolC components, inhibiting the overall assembly and function of the efflux pump by altering its conformational changes (Sharma et al., 2019). Among these, the recently developed BDM series of pyridine-piperazine compounds, such as BDM91288, can specifically bind to key sites in the AcrB structure, significantly blocking the AcrAB-TolC efflux system in *K. pneumoniae*, showing good synergistic effects with quinolones and other antibiotics, and demonstrating excellent oral bioavailability and pharmacokinetic properties (Vieira Da Cruz et al., 2024). This precise targeting mechanism effectively reduces non-specific toxicity risks and improves the selectivity and clinical potential of the drug.

Although EPI development faces numerous challenges such as compound permeability, toxicity, metabolic stability, and the risk of resistance development, the continuous deepening of our understanding of efflux pump structures and functional mechanisms, along with the rapid development of computational-aided drug design and high-throughput screening technologies, is making the discovery and optimization of new-generation EPIs increasingly possible. Future research should focus on addressing the issue of poor *in vivo* and *in vitro* efficacy of EPIs and explore optimized strategies for combination with novel antimicrobial drugs to facilitate the true clinical translation of EPIs.

## 7 Physicochemical methods

In addition to targeting specific biochemical pathways to combat Gram-negative bacteria, researchers have also explored optimizing the physicochemical properties of drug molecules to enhance their ability to penetrate the outer membrane or assisting drug delivery systems in crossing this barrier. These strategies do not directly target specific bacterial enzymes or metabolic pathways but instead modulate the physicochemical interactions, such as charge, polarity, hydrophobicity, and porin affinity between drugs and the outer membrane, thereby increasing intracellular drug concentrations and achieving antimicrobial effects.

### 7.1 Drug design to optimize outer membrane permeation based on physicochemical properties

The outer membrane of Gram-negative bacteria acts as a formidable selective barrier, significantly restricting the permeability of most antibiotics. Therefore, optimizing the physicochemical properties of drug molecules to improve their ability to penetrate the outer membrane and accumulate inside the cell has become a key strategy for enhancing antimicrobial activity (Cooper et al., 2018). This strategy represents a fundamental exercise in rational molecular design aimed at bypassing the specific physicochemical constraints imposed by the outer membrane architecture. To be effective, antibiotic molecules must navigate two primary biophysical hurdles: the strong electrostatic repulsion generated by the anionic LPS layer and the stringent steric restrictions of the narrow porin channels. In this context, the eNTRy rules proposed by Haloi et al. (2021) provide a seminal roadmap for overcoming these obstacles. Although initially established based on *E. coli* permeability data, the eNTRy framework highlights key physicochemical features that guide drug molecules efficiently into Gram-negative bacteria, even if its applicability may vary among species (Haloi et al., 2021). These features include: the presence of an ionizable amine group, which facilitates electrostatic interactions with the negatively charged LPS core oligosaccharide region; low globularity, which makes the drug more linear or flattened in structure, thereby enhancing its ability to traverse porin channels; and higher molecular rigidity, indicated by fewer rotatable bonds, which reduces entropy loss during transmembrane diffusion, thermodynamically favoring permeation.

These design principles have been successfully applied in the development of new antimicrobial drugs. A typical example is the new generation broad-spectrum fluoroquinolone delafloxacin, where the molecule’s rigidity and charge distribution were specifically optimized to improve its permeability and antimicrobial activity against various resistant Gram-negative bacteria, such as *P. aeruginosa* and *E. coli* (El-Sagheir et al., 2025). Another classic example is the structural optimization of deoxynybomycin. Deoxynybomycin, a natural DNA gyrase inhibitor, was originally only effective against Gram-positive bacteria but had difficulty penetrating the outer membrane of Gram-negative bacteria (Lewis, 2020). Based on the eNTRy rule, Gurvic and Zachariae (2024) introduced a primary amine group into the molecule’s structure, enhancing its hydrophilicity and positive charge without affecting its target binding ability, thereby expanding its antimicrobial spectrum to include Gram-negative bacteria and demonstrating the tremendous potential of rational drug design.

However, it should be noted that the permeability of the Gram-negative bacterial outer membrane is influenced by multiple factors. Differences in the LPS structure, porin expression, and membrane permeability across different species result in variability, meaning the universality of the eNTRy rule may differ between strains or species (Saxena et al., 2023). Additionally, other physicochemical features such as the molecular polar surface area, hydrogen bond donor/acceptor count, and dipole moment also play significant roles in drug transmembrane permeability (Gurvic and Zachariae, 2024). Furthermore, after crossing the outer membrane, antimicrobial molecules may become substrates for efflux pumps, thereby limiting intracellular accumulation and increasing uncertainty in their antimicrobial efficacy. Therefore, future drug design must simultaneously optimize drug permeability and its ability to evade recognition by efflux pumps. To achieve this, high-throughput screening technologies and computational methods should be utilized to quantitatively analyze the specific outer membrane composition characteristics of different bacterial species and their impact on drug permeation, thus guiding the design of personalized antimicrobial drugs to improve drug development success rates and clinical efficacy. In summary, the permeability of the Gram-negative bacterial outer membrane results from a synergy of multiple factors, and the development of antimicrobial drugs should consider factors such as drug molecule size, polarity, charge distribution, rigidity, and efflux pump substrate recognition features to effectively enhance drug accumulation within bacteria and therapeutic efficacy.

### 7.2 Nanocarrier systems

In addition to optimizing the physicochemical properties of the drug itself, the development of novel drug delivery systems using nanotechnology has become a cutting-edge strategy for enhancing antibiotic activity, especially for drugs with poor intrinsic permeability or those prone to degradation (Makabenta et al., 2021). Nanocarriers can encapsulate antibiotics within their core or adsorb them onto their surface, significantly improving the drug’s bioavailability by enhancing its solubility, stability, and release kinetics, while protecting it from bacterial enzymatic degradation (Ahsan et al., 2024). Moreover, nanocarriers can substantially increase the local concentration of drugs on the bacterial surface and, through special mechanisms such as membrane fusion, endocytic pathways, or localized disruption of the outer membrane structure, further promote drug permeation across the outer membrane of Gram-negative bacteria, thereby greatly enhancing the antimicrobial effect. Currently, various nanomaterials, including liposomes, polymeric nanoparticles, solid lipid nanoparticles, nanostructured lipid carriers, metallic nanoparticles, and carbon-based nanomaterials, have been widely explored for antimicrobial drug delivery (Su and Kang, 2020).

The mechanisms of action of nanocarriers are diverse. By altering the overall physicochemical properties of the drug, such as increasing its solubility and stability, nanocarriers can significantly improve the drug’s bioavailability. Many nanocarriers possess the ability to strongly interact with bacterial outer membranes (Modi et al., 2023). For example, cationic liposomes or polymeric nanoparticles adsorb onto the negatively charged LPS on the bacterial outer membrane through electrostatic interactions, leading to local disruption or even destruction of the membrane structure, which improves the drug’s transmembrane permeability. In some cases, this membrane-damaging mechanism of the nanocarriers themselves may also exert antimicrobial effects. Additionally, nanocarriers can be surface-modified with specific ligands, such as short peptides or small molecules that bind selectively to outer membrane porins, achieving a “Trojan horse” style active delivery mechanism, inducing bacterial uptake of drug-loaded nanoparticles (Huang et al., 2013). These carriers also protect the drugs from degradation by outer membrane and intracellular enzymes, and by increasing the intracellular drug concentration or local enrichment, they effectively reduce efflux pump-mediated drug expulsion, significantly improving drug penetration and distribution in complex infection environments, such as biofilms. Furthermore, through precise design, nanocarriers can achieve targeted responses to specific infection microenvironments, enabling targeted drug release and minimizing the non-specific toxicity to host cells. For example, research has developed polymeric nanocarrier systems capable of simultaneously loading multiple antibiotics, which release drugs upon contact with bacteria, achieving a multi-target synergistic effect and significantly enhancing antimicrobial efficacy (Meng et al., 2025).

## 8 Conclusion and future directions

**FIGURE 2:**
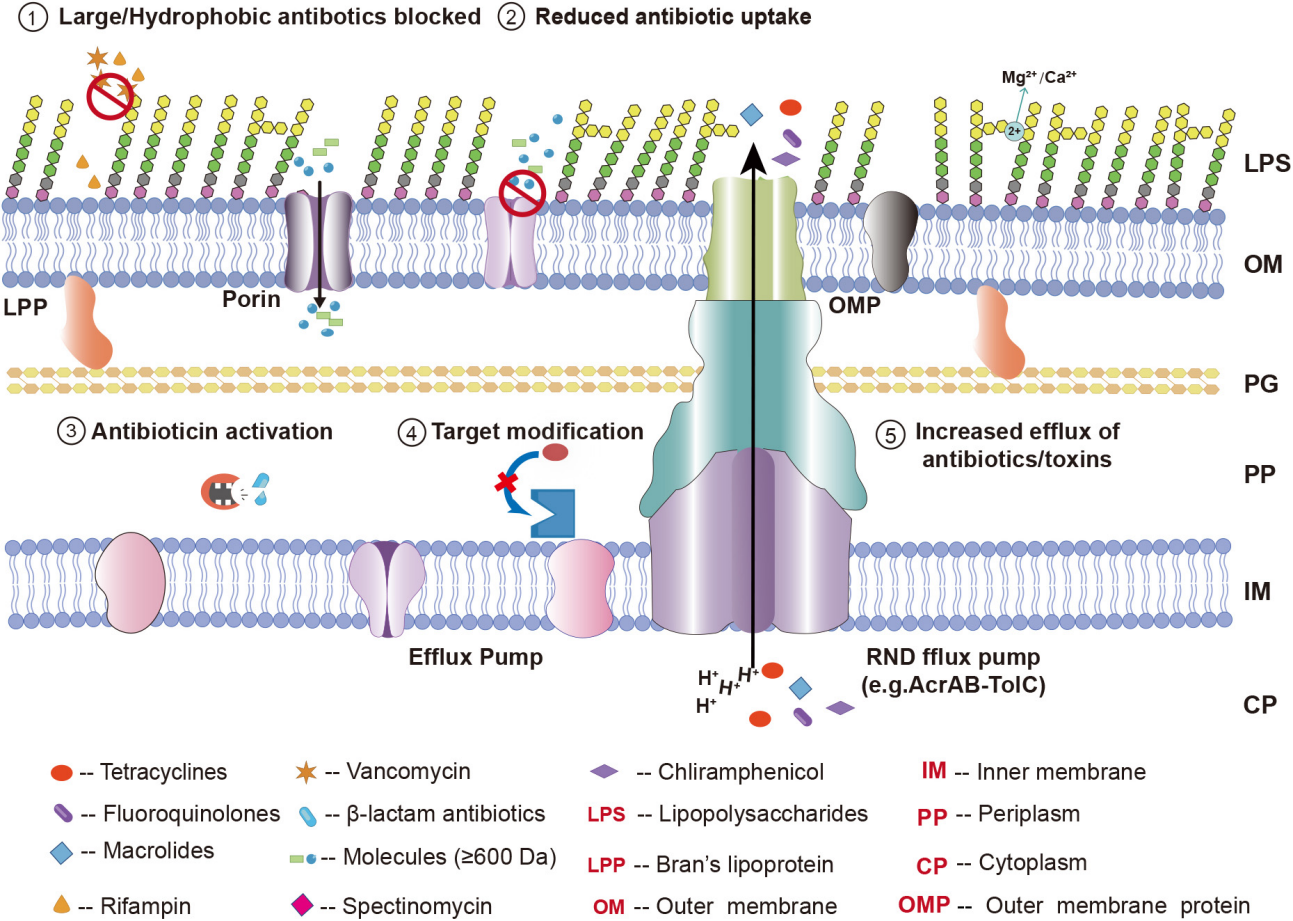
The multilayer intrinsic resistance mechanisms of the Gram-negative bacterial cell envelope. The schematic illustrates the sequential barriers that contribute to multidrug resistance. (1) The asymmetric outer membrane, rich in LPS, acts as a formidable permeability barrier, blocking the entry of large and/or hydrophobic antibiotics. (2) Small hydrophilic antibiotics primarily cross the outer membrane via porins. Mutations or downregulation of porins can significantly reduce antibiotic uptake. (3) Once in the PP, antibiotics such as β-lactams can be hydrolyzed and inactivated by enzymes like β-lactamase. (4) Antibiotics that reach their CP targets may be ineffective due to target site modifications. (5) A major resistance mechanism is the active efflux of diverse antibiotics from the cell interior and periplasm by powerful tripartite efflux pumps, such as the RND-type AcrAB-TolC system, which spans the entire cell envelope. These collaborative mechanisms ensure that even if an antibiotic penetrates one line of defense, it can be neutralized by another, leading to high-level intrinsic resistance.

**FIGURE 3:**
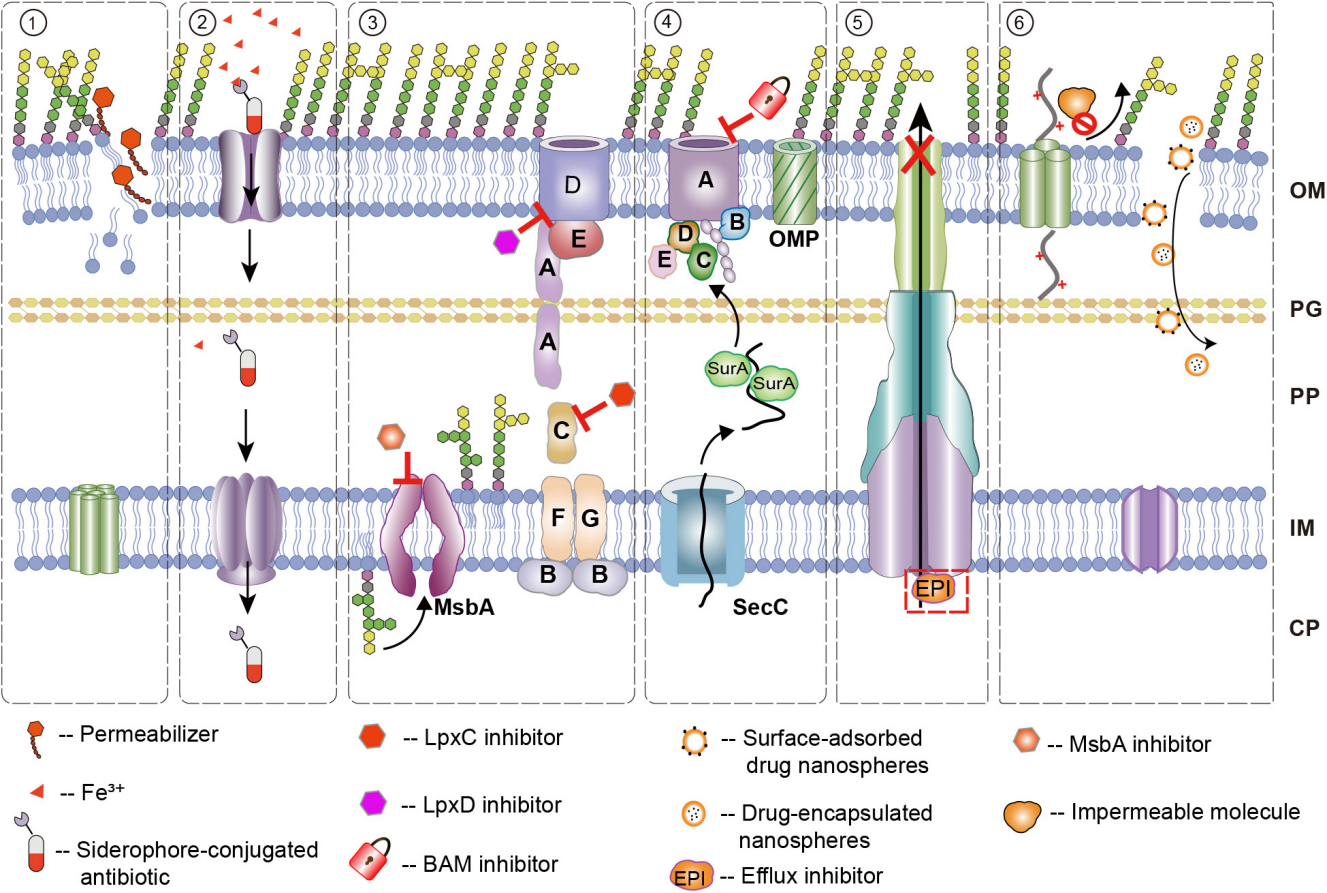
Multifaceted antimicrobial strategies to overcome the outer membrane barrier of Gram-negative bacteria. The illustration summarizes the key strategic approaches discussed in this review, which are designed to combat Gram-negative bacteria by targeting their formidable outer membrane: (1) direct membrane disruption. Permeabilizers such as polymyxins, CAMPs, and chelators directly compromise the integrity of the asymmetric outer membrane by interacting with LPS, leading to increased permeability and cell death. (2) Hijacking natural uptake pathways. This strategy exploits the bacteria’s own nutrient import systems. Siderophore-antibiotic conjugates act as “Trojan horses,” being actively imported via TonB-dependent receptors. (3, 4) Inhibition of biosynthesis and assembly. This approach targets the biogenesis of key outer membrane components. It includes inhibitors of LpxC and the MsbA/Lpt systems to block LPS synthesis and transport, and BAM complex inhibitors to prevent OMP assembly. (5) Targeting efflux pumps. EPIs block the activity of multidrug efflux pumps. By inhibiting drug extrusion, EPIs increase the intracellular concentration of antibiotics, thereby resensitizing resistant strains. (6) Physicochemical methods and nanocarrier systems. This includes the rational design of drug molecules (e.g., following the eNTRy rules) to enhance outer membrane penetration, drug design optimized for porin-mediated diffusion, and leveraging the outer membrane’s Donnan potential. Additionally, the use of drug-loaded nanocarriers that adsorb to or fuse with the outer membrane further improves drug delivery and efficacy.

The outer membrane of Gram-negative bacteria acts as a formidable barrier, playing a pivotal role in multidrug resistance due to its asymmetric structure, low permeability, and multifunctionality. As summarized in Figure 2, even if a drug successfully penetrates the outer membrane, it may still be inactivated in the periplasm, fail to bind to a modified target, or be effectively extruded by powerful efflux pumps. This collaborative system ensures a high level of intrinsic and acquired multidrug resistance in Gram-negative pathogens. Recent advances have shifted the focus from merely disrupting membrane permeability to targeting complex processes such as component synthesis, transport, and homeostasis. To provide a cohesive framework for these emerging paradigms, Figure 3 systematically categorizes the multifaceted strategies discussed throughout this review. Furthermore, Table 1 complements this visual overview by summarizing the mechanisms of action, representative compounds, and current developmental status of these innovative antimicrobial agents.

**TABLE 1:** Summary of antimicrobial strategies targeting the outer membrane of Gram-negative bacteria.

Strategy category	Representative molecule/type	Molecular target	Mechanism of action	Key features/limitations	Development stage
Disrupting outer membrane integrity	Polymyxin B/E (colistin)	LPS (lipid A)	Displaces divalent cations, disrupts LPS layer structure, increases membrane permeability	Nephrotoxicity and neurotoxicity limit use; last-resort antibiotic	Clinical use
	PMBN (polymyxin B nonapeptide)	LPS	Deacylated derivative, retains membrane permeability-enhancing ability, minimal direct antibacterial activity	Research tool for studying outer membrane permeability; potentiates other antibiotics	Research tool/preclinical
	Cationic antimicrobial peptides (e.g., LL-37)	Entire outer/inner membrane	Forms transmembrane pores, membrane depolarization, multi-target action	Immunomodulatory properties; potential resistance development	Preclinical research
Inhibiting LPS biosynthesis	ACHN-975	LpxC	Inhibits UDP-3-O-(acyl)-GlcNAc deacetylase, blocks lipid A synthesis	Poor pharmacokinetics led to termination	Phase I (terminated)
	LPC-233, TP0586532	LpxC	Same mechanism as ACHN-975; structural optimization for improved PK/PD	Next-generation LpxC inhibitors with improved properties	Preclinical research
Inhibiting LPS transport	Murepavadin (POL7080)	LptD	Blocks LPS insertion into outer membrane, causes precursor accumulation	*P. aeruginosa*-specific; renal toxicity led to suspension	Phase III (suspended)
	Zosurabalpin (RG6006)	LptB2FGC	Inhibits LPS transport ATPase, blocks LPS transmembrane transport	First-in-class for CRAB; *Acinetobacter*-specific activity	Phase I (Phase III planned 2025–2026)
Inhibiting OMP assembly	Darobactin A/darobactin 22	BamA	Inhibits BamA lateral gate dynamics, interferes with β-barrel protein folding and insertion	Novel bicyclic peptide structure; active against MDR *E. coli*, *K. pneumoniae*, *P. aeruginosa*	Preclinical research
	Xenorceptides	BamA	Induces BamA structural remodeling, completely closes lateral gate channel	Distinct binding mode from darobactin	Preclinical research
	JB-95	BamA/LptD	Dual-target inhibition, interferes with OMP assembly and LPS transport	Broad-spectrum activity; potential advantage of dual mechanism	Preclinical research
Inhibiting lipoprotein maturation and localization	Globomycin	LspA (lipoprotein signal peptidase II)	Inhibits lipoprotein signal peptidase, blocks lipoprotein maturation	Natural product from *Streptomyces*; synergizes with β-lactams	Preclinical research
	Myxovirescin	Lnt (lipoprotein N-acyltransferase)	Inhibits lipoprotein N-acylation, prevents complete triacylation	Essential for outer membrane stability; potential synergy with other agents	Preclinical research
	MAC13243	LolA/LolCDE	Inhibits lipoprotein outer membrane localization system, causes abnormal accumulation	Disrupts lipoprotein trafficking pathway	Preclinical research
	Lolamicin	LolCDE complex	Inhibits lipoprotein transport to outer membrane	Microbiome-sparing: Selectively kills pathogenic Gram-negatives while preserving gut commensals; active against MDR strains	Preclinical research
Utilizing natural pathways	Cefiderocol	TonB-dependent siderophore receptors	“Trojan horse” strategy, active uptake through siderophore system	FDA-approved 2019; active against MDR/XDR pathogens including CRAB	Clinical use
	BAL30072	Siderophore receptor	Monobactam conjugated with hydroxypyridone, mimics siderophore	Synthetic siderophore-antibiotic conjugate	Preclinical research
Inhibiting efflux pumps	PAβN (phenylalanine-arginine β-naphthylamide)	RND efflux pump (e.g., AcrB)	Competitive substrate inhibitor, blocks drug efflux	Non-specific; used as research tool; toxicity concerns	Research tool
	BDM91288	AcrB	Specifically binds to functional site of pump protein, inhibits substrate efflux	Improved specificity over PAβN	Preclinical research
Physicochemical optimization	Delafloxacin	Outer membrane porins	Optimizes charge, rigidity and molecular size based on eNTRy rules	Weakly acidic fluoroquinolone; approved 2017 for ABSSSI	Clinical use
Nanocarrier systems	Liposomes, polymeric nanoparticles, metal nanoparticles (e.g., Ag, Au)	Outer membrane structure	Enhances drug delivery through membrane fusion, electrostatic interactions or active targeting	Versatile platform for drug delivery; potential for combination therapy	Preclinical/early clinical research

Some strategies have significantly enhanced antimicrobial activity by directly damaging the outer membrane or increasing drug permeability. However, these often cause high toxicity due to non-specific damage to host membrane stability and ion balance, limiting clinical applications. In contrast, strategies targeting the synthesis and transport of outer membrane components exhibit greater selectivity and innovation. For instance, BAM complex inhibitors like darobactin and xenorceptides specifically interfere with OMP assembly and show good bactericidal effects against Gram-negative bacteria, such as Enterobacteriaceae and *P. aeruginosa*. Lpt system inhibitors, such as murepavadin and zosurabalpin, disrupt outer membrane integrity by blocking LPS transport. However, they exhibit significant toxicity and pharmacokinetic defects in preclinical studies, with murepavadin showing nephrotoxicity in phase III clinical trials. Similarly, inhibitors targeting the key enzyme in LPS synthesis, LpxC, such as ACHN-975, despite demonstrating good antimicrobial activity *in vitro*, failed in clinical stages due to significant host toxicity. These failures highlight the bottlenecks in new drug development, such as insufficient selectivity, target discrepancies, and differences between *in vitro* and *in vivo* effects.

Moreover, the “Trojan Horse” strategy, which utilizes natural transport systems to facilitate drug entry into cells, has successfully translated into clinical applications. For example, siderophore-conjugated antibiotics like cefiderocol have shown significant advantages in treating resistant Gram-negative bacterial infections. However, long-term use may induce new resistance mechanisms. Future research needs to systematically monitor bacterial adaptation to siderophore-conjugated drugs and identify and prevent potential resistance risks through directed evolution experiments and genome sequencing. EPIs show promise as adjunctive treatments to enhance antibiotic efficacy. However, traditional EPIs, such as PAβN, face significant challenges in clinical translation due to issues with pharmacokinetics, including poor bioavailability and rapid clearance, as well as toxicity concerns. These obstacles highlight the need for further optimization of EPI-based strategies. Beyond small-molecule strategies, the therapeutic application of bacteriophage-derived enzymes, referred to as enzybiotics, represents a burgeoning strategy to breach the Gram-negative envelope. Depolymerases, typically derived from phage tailspike proteins, specifically degrade capsular polysaccharides and LPS, thereby compromising bacterial defense mechanisms (Zhang et al., 2025). Endolysins offer a distinct mechanism by cleaving the peptidoglycan layer. The fusion of endolysins with cationic peptides, resulting in Artilysins, enables effective penetration of the outer membrane barrier (Briers et al., 2014). Despite their promise, several hurdles impede clinical translation, including enzyme instability, potential immunogenicity, and the need for optimal delivery systems. Future success will depend on overcoming these limitations to realize their full therapeutic potential.

In recent years, despite significant progress in the development of antimicrobial drugs targeting the outer membrane, frequent failures in clinical trials indicate the need to reflect deeply on the fundamental causes of these failures. Firstly, the target selection and off-target effects of failed drugs should be carefully examined. For example, the clinical failure of LpxC inhibitors was due to the hydroxamic acid group, which not only inhibits the bacterial target but also non-specifically binds to metal enzymes in host cells, leading to unpredictable toxicity. Secondly, although EPIs perform well *in vitro*, their *in vivo* efficacy is greatly diminished, possibly due to host drug metabolism inactivation, high serum protein binding, and dynamic changes in efflux pump expression in the infectious microenvironment. Additionally, bacteria may weaken the effect of a single EPI through redundancy or compensatory mechanisms between similar efflux pumps. Therefore, there is an urgent need to develop more realistic preclinical models that simulate real infection environments and establish accurate pharmacokinetic-pharmacodynamic prediction platforms.

To address these bottlenecks, future research should be more focused, concentrating on specific scientific issues. Artificial intelligence and MD simulation techniques should be used to construct fine transmembrane permeability prediction models, which could enhance the efficiency of drug development. Preclinical infection models should better reflect human physiological conditions. For example, using human-derived lung, intestinal, or bladder organoid infection models can evaluate drug efficacy and toxicity under more human-like physiological and pathological conditions. At the same time, these models should be validated against traditional animal models or cell culture systems to ensure that the new models can accurately predict clinical translation potential. In parallel, antimicrobial discovery is likely to benefit from a shift beyond single-target paradigms toward rationally engineered, multi-target combinations. In Gram-negative pathogens, the fortified outer membrane and diverse resistance mechanisms function as an integrated defense network, implying that coordinated disruption may be required for durable activity, such as combining BAM complex inhibitors with LPS biosynthesis inhibitors, which could simultaneously impair outer membrane protein insertion and LPS replenishment. Such strategies have the potential to induce irreversible structural damage to the bacterial envelope, offering new opportunities to overcome resistance. Future research should also focus on the precise development of antibiotics, systematically studying the structural differences in target sites between pathogens and host microbiota, and designing microbiota-friendly “smart” inhibitors that maximize infection clearance while protecting the host microbiota.

In conclusion, antimicrobial strategies targeting the outer membrane of Gram-negative bacteria are at a crucial and complex stage of development. Future breakthroughs will depend not only on the discovery of new antimicrobial molecules but also on continuous in-depth research from basic mechanism studies and preclinical efficacy evaluations to resistance prevention, as well as interdisciplinary integration. This will bring revolutionary progress to antimicrobial drug development and help us play an active role in the long-term fight against bacterial resistance.
